# Isolated hepatoblastoma arising from the hepatogastric ligament: a case report

**DOI:** 10.1186/s13256-017-1488-8

**Published:** 2017-11-21

**Authors:** Ji Chen, Mengjiao Sun, Bin Sun, Jun Yi, Bin Jiang, Lei Huang

**Affiliations:** 1 Department of General Surgery, Children’s Hospital of Nanjing Medical University, Nanjing, 210008 China; 2 Department of Hematology/Oncology, Children’s Hospital of Nanjing Medical University, Nanjing, China

**Keywords:** Hepatoblastoma, Hepatogastric ligament, Extrahepatic, Rare

## Abstract

**Background:**

Almost all hepatoblastomas are isolated to the liver. Hepatoblastoma arising from and limited to extrahepatic tissue is an extremely rare clinical entity.

**Case presentation:**

Here we present a case of a 7-year-old Chinese boy of Han ethnicity with hepatoblastoma originating from the hepatogastric ligament. A complete resection was performed and the entire course was uneventful. He received six cycles of postoperative chemotherapy and had no signs of recurrence for 3 years after surgery.

**Conclusions:**

Hepatoblastoma arising from extrahepatic tissue is extremely rare. A pedunculated hepatoblastoma is prone to hemorrhage and tumor metastasis. The best treatment for a long-term cure is complete resection of the primary tumor combined with chemotherapy.

## Background

Primary pediatric hepatic malignancies occupy 1 to 2% of all pediatric tumors. Arguably, the most common histological subtype of primary malignant hepatic tumors in children is hepatoblastoma, which comprises the plurality of liver malignancies at a 79% incidence [1, 2]. Almost all hepatoblastomas are isolated to the liver. Hepatoblastoma arising from extrahepatic tissue is extremely rare.

## Case presentation

**Fig. 1:**
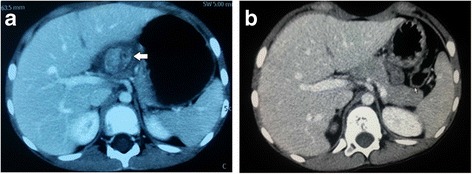
**a** Contrast-enhanced abdominal computed tomography showed there was a mass in the hepatic portal area (*left arrow*), the size of which was approximately 3.2 × 4.3 cm, and it slightly adhered to the surrounding tissue. The mass was not uniformly enhanced, the center of the tumor had obvious enhancement, and there was no significant invasion of our patient’s liver. **b** Contrast-enhanced abdominal computed tomography after operation

**Fig. 2:**
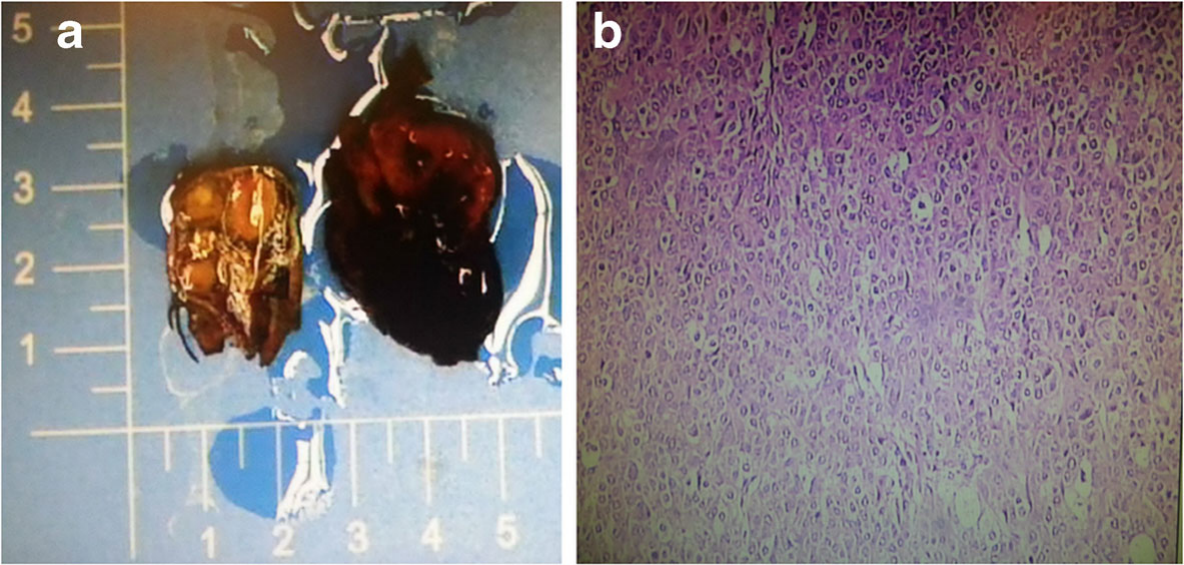
**a** The postoperative specimen was dark red with a size of 2.5 × 3.5 cm and it had a complete capsule. The cut surface of this tumor had a soft, hemorrhagic necrotic and yellow nodular appearance. **b** Pathology showed hepatoblastoma in the hepatogastric ligament (subtype, mixed fetal and embryonal hepatoblastoma)

A 7-year-old Chinese boy of Han ethnicity presented to our Department of General Surgery, Children’s Hospital of Nanjing Medical University with an abdominal mass that was revealed on ultrasound examination after a tumble. His serum alpha-fetoprotein (AFP) was elevated to 163.1 ng/ml (normal, 8.5 ± 5.5 ng/ml). An abdominal computed tomography (CT) showed that there was an inhomogeneous low density mass in the hepatic portal area, the size of which was approximately 3.2 × 4.3 cm, and it slightly adhered to the surrounding tissue. Contrast-enhanced CT showed that the mass was not uniformly enhanced, the center of the tumor had obvious enhancement, and there was no significant invasion of his liver (Fig. 1a). An abdominal examination showed his abdomen was soft, there was no gastrointestinal peristalsis, no obvious abdominal mass, and no abdominal tenderness and rebounding pain. Bowel sounds were normal. An exploratory laparotomy was performed after preoperative examination and an abdominal approach through abdominal median incision was chosen. A mixed mass of approximately 4 × 3 cm was found in the hepatogastric ligament. The mass was not attached to large vessels and was nourished by the capillary vessels in the hepatogastric ligament. There was a small amount of bleeding in the mass, which had complete tumor capsule and adhered mildly to the lesser omentum. We removed the tumor completely after loosening it from surrounding tissue. The operation was performed successfully and the intraoperative diagnosis was teratoma. The postoperative specimen showed: specimen was dark red with a size of 2.5 × 3.5 cm and it had a complete capsule. The cut surface of this tumor had a soft, hemorrhagic necrotic and yellow nodular appearance (Fig. 2a). Pathology showed hepatoblastoma in the hepatogastric ligament: subtype, mixed fetal and embryonal hepatoblastoma (Fig. 2b). Immunohistochemistry showed: creatine kinase (CK), (+); epithelial membrane antigen (EMA), (+); glypican-3, (+); human chorionic gonadotropin (HCG), (−); vimentin, (−); S100, (−); cluster of differentiation (CD) 34, (−); and Ki-67, (40%+). His AFP dropped to 18.33 ng/ml on the 13th day and returned to normal on the 28th day after the operation. He underwent six cycles of postoperative chemotherapy. There was no sign of recurrence through abdominal CT in the 3 years after surgery (Fig. 1b).

## Discussion

Most hepatoblastomas occur before the age of 3 years, with a median age of approximately 18 months, and they account for 58% of the malignancies diagnosed in children with extremely low birthweight [3, 4]. Hepatoblastomas tend to be unifocal lesions in most cases: 50% isolated to the right lobe, 15% to the left lobe, and 27% centrally located to involve both lobes. The best treatment for a long-term cure is complete resection of the primary tumor combined with chemotherapy. The age of our patient was beyond the usual age of development of hepatoblastoma. According to our patient’s medical history, he was initially diagnosed as having abdominal hematoma. We found that the tumor looked like a teratoma during the operation. However, pathology and immunohistochemistry showed that it was a hepatoblastoma in the hepatogastric ligament. Hepatoblastoma arising from extrahepatic tissue is extremely rare. We found only one report on hepatoblastoma that was pedunculated from the right lobe [5] and several reports of hepatocellular carcinoma arising from ectopic liver tissue [6–9]. An isolated hepatoblastoma in the hepatogastric ligament has not been reported before in PubMed.

We could better understand a pedunculated hepatoblastoma through our learning of pedunculated hepatocellular carcinoma [10, 11]. Pedunculated hepatocellular carcinoma is divided into two types: pedicle type and non-pedicle type. The pedunculated tumor pedunculated from the right lobe of the liver is more common and the majority of non-pedicle types are organized from the left lobe. A pedunculated tumor is connected with the liver by the pedicle which transports nutrition from the liver. After the invasion of the surrounding tissue, the growth rate of the tumor is obviously accelerated because of the establishment of a new blood supply. In contrast to the pedicle type, a small part of a non-pedicle-type tumor is located in the liver parenchyma and the majority is located outside the liver. In our rare case, the tumor was isolated in the hepatogastric ligament without any pedicles from the liver. Without the protection of liver parenchyma, the pedunculated hepatocellular carcinoma was prone to hemorrhage, which would easily lead to tumor metastasis. In this case, we found the intratumoral hemorrhage, but the tumor was completely wrapped by a capsule and was completely resected. At present, six cycles of chemotherapy have been completed and there are no signs of recurrence. However, a longer follow-up is necessary to assess the prognosis of this child in future.

## Conclusions

This is to the best of our knowledge a rare case of isolated hepatoblastoma arising from extrahepatic tissue. Extrahepatic hepatoblastoma is prone to hemorrhage and tumor metastasis. The best treatment for a long-term cure is complete resection of the primary tumor combined with chemotherapy.

## Abbreviations

AFP: Alpha-fetoprotein
CT: Computed tomography
